# Age, Gender, and Disease Duration as Key Determinants of Comorbidity Burden in Spondyloarthritis: A Multicentre Cross-Sectional Study

**DOI:** 10.31138/mjr.230525.dkr

**Published:** 2025-12-31

**Authors:** Chandrashekara S, Padmanabha Shenoy, Uma Kumar, Sapan Pandya, Alakendu Ghosh, Apurva Khare, Rajkiran Dudam, Rudra Prosad Goswami

**Affiliations:** 1 ChanRe Rheumatology & Immunology Centre & Research, Bengaluru, Karnataka, India;; 2 Centre for Arthritis & Rheumatism Excellence (CARE), Cochin, Kerala, India;; 3 All India Institute of Medical Science, New Delhi, India;; 4 Rheumatic Disease Clinic, Vedanta Institute of Medical Science, Ahmedabad, Gujarat, India;; 5 Clinical Immunology & Rheumatology, Institute of Post-Graduate Medical Education and Research, Kolkata, India;; 6 Department of General Medicine, LN Medical College and Research Centre, Madhya Pradesh, India;; 7 HRC Hospital, Prakash Nagar, Begumpet, Hyderabad, India;; 8 Department of Rheumatology, AIIMS, New Delhi, India

**Keywords:** spondyloarthritis, axial spondyloarthritis, comorbidity, diabetes mellitus, hypertension, thyroid diseases

## Abstract

**Objective:**

The burden of comorbidities associated with spondylarthritis (SpA) in India remains relatively unexplored, with most existing research limited to specific regions. This study aimed to evaluate the prevalence and patterns of comorbidities among SpA patients across India.

**Methods:**

This multicentre, observational study was conducted at seven centres across India using data from the Indian Rheumatology Association database. Comorbidities were classified according to the ICD-10 Charlson Comorbidity Index. Patients were stratified into two age groups (>50 vs. ≤50 years). Statistical analyses included descriptive statistics, Fisher’s exact test, chi-square test, two-tailed t-test, and logistic regression to identify predictors of comorbidity.

**Results:**

Of 1,250 SpA patients (mean age 39.8 ± 13.3 years), 25% had comorbidities. The most common were hypertension (11.8%) and diabetes (8.5%), including 1.1% with complications. Comorbidities were significantly more frequent in patients >50 years (55.3%) vs ≤50 years (16.6%, P < 0.001). Older age was associated with higher rates of diabetes (24.5% vs. 4.0%), hypertension (32.6% vs. 6.0%), and thyroid disorders (9.5% vs. 2.8%) (P < 0.001 for all). Logistic regression revealed age as the strongest predictor for hypertension (P < 0.001, Wald = 101.3), diabetes (P < 0.001), hyperlipidaemia (P = 0.022), and thyroid disorders (P = 0.003). Female gender was associated with thyroid disorders (P < 0.001), and longer disease duration with diabetes (P = 0.022).

**Conclusion:**

This study underscores a substantial comorbidity burden among Indian SpA patients, highlighting the need for comprehensive screening and management strategies, particularly in older patients and those with longer disease duration.

## INTRODUCTION

Spondyloarthritis (SpA) refers to a group of inflammatory diseases that includes ankylosing spondylitis (AS), reactive arthritis, psoriatic arthritis (PsA), inflammatory bowel disease-associated SpA, and undifferentiated SpA.^1,2^ The prevalence of SpA varies widely across regions and ethnic groups due to factors such as population heterogeneity and differences in study methodologies.^3^ In India, epidemiological studies estimate its prevalence at approximately 7–9 cases per 10,000 individuals.^2^

Comorbidities in inflammatory rheumatic disorders such as SpA are associated with increased morbidity and mortality, reduced functional capacity, and poorer patient-reported outcomes. Furthermore, comorbidities can affect medication tolerability and may influence decisions regarding the use of biological therapies.^4^ In SpA, extra-articular manifestations and comorbidities contribute to greater disability and higher healthcare costs.^5^ The Assessment of SpondyloArthritis International Society (ASAS) COMOrbidities in SpondyloArthritis (COMOSPA) study has identified key comorbidities associated with SpA, including cardiovascular diseases, osteoporosis, cancers, infections, and gastrointestinal disorders. Additionally, the study highlights specific risk factors contributing to the development of cardiovascular diseases, cancers, and osteoporosis in these patients.^6^

A nationwide population-based study by Redeker et al. noted that the presence of comorbidities in SpA patients is associated with increased disease activity and functional impairment.^7^ Comorbidities significantly influence therapeutic decisions, treatment outcomes, and overall disease management. These concurrent conditions can alter the course of SpA, affect the efficacy and tolerability of therapeutic interventions, especially biologic agents, and complicate clinical decision-making. Furthermore, comorbidities have a profound impact on physical function, health-related quality of life, and healthcare utilisation.

The burden of SpA-related comorbidities in India remains relatively underexplored, with existing studies largely limited to specific geographical regions. This multi-center study aims to analyse data from the Indian Rheumatology Association (IRA) database to assess the prevalence and patterns of comorbidities among SpA patients across the country. By evaluating both clinical parameters and demographic characteristics, the findings are expected to aid healthcare providers in risk stratification, treatment planning, and long-term monitoring, thereby assisting in developing more effective and personalised management strategies for SpA.

## MATERIALS AND METHODS

### Study Design and Setting

This multicentre, cross-sectional observational study was conducted at seven geographically diverse rheumatology centres across India. The study used data from the Indian Rheumatology Association (IRA) registry, established in April 2020 to collect information on patients with six autoimmune rheumatic diseases (AIRDs): rheumatoid arthritis (RA), spondyloarthritis (SpA), psoriatic arthritis (PsA), systemic lupus erythematosus (SLE), systemic sclerosis, and primary Sjögren’s syndrome (pSS).

### Study participants

Adult patients (≥18 years), including newly diagnosed and follow-up cases, who fulfilled the Assessment of SpondyloArthritis International Society (ASAS) classification criteria for SpA were included.^6^ The final patient was enrolled in June 2022.

### Data collection

Two structured, expert-validated forms were used to collect data: one capturing general demographics and clinical information, and the other focusing on disease-specific parameters. Clinical research associates (CRAs) completed these forms based on interviews and medical record reviews. To ensure uniformity, all principal investigators (PIs) and CRAs participated in online training sessions, with the central centre’s PI addressing any data collection queries.

### Ethical approval

Ethical approval was obtained from the Institutional Ethics Committees and Independent Ethics Committees of all participating centres, and written informed consent was obtained from all patients. Approvals were granted under the following protocol numbers and approval dates: IEC-CRICR-132/101/2020 (05/10/2020), IEC-59/03.07.2020.RP-462020 (15/07/2020), IPGME&RC/IEC/2020/478 (16/06/2020), and LNMC&RC/Dean/2020/Ethics/146 (07/08/2020) (Details are provided in the supplementary table.)

### Sample size estimation

The minimum number of subjects with autoimmune inflammatory rheumatic diseases (AIRDs) needed for the registry was determined to allow for meaningful comparisons. The focus was on evaluating the clinical and laboratory profiles of the seven AIRDs being studied. The estimated total sample size for analysis was set at 6,500 patients.^8^ The sample size for each disease was determined based on the prevalence of each autoimmune condition, with a proportionate allocation for each. For SpA, with an estimated prevalence ranging from 0.07% to 0.09%, a minimum target of approximately 700 individuals was established.^2^

### Data definition and comorbidity assessment

Comorbidities were defined using the latest ICD-10-based Charlson Comorbidity Index and Information was obtained through patient interviews and medical chart reviews.^9^ Comorbidities were verified through past evaluations and/or treating physician reports, and trained site-specific experts performed systematic assessments using ICD codes.^10^

Notable comorbidities included thyroid disorders (hypothyroidism/hyperthyroidism), diabetes (with and without complications), cerebrovascular events (stroke, transient ischemic attack), psychiatric conditions (including neurosis and depression), infectious diseases (such as tuberculosis and hepatitis B/C), myocardial infarction, congestive heart failure, congenital heart disease, rheumatic heart disease, peripheral vascular disease, migraine, hemiplegia, dementia, Parkinson’s disease and other degenerative neurological disorders, chronic pulmonary disease, bronchial asthma, rheumatologic diseases, psoriasis, osteoporosis, fibromyalgia, osteoarthritis, peptic ulcer disease, recurrent diarrhoea of unknown cause, inflammatory bowel disease (IBD), mild liver disease, gout, hyperlipidaemia, moderate-to-severe renal disease, non-metastatic cancer, leukemia, lymphoma, moderate or severe liver disease, metastatic solid tumours, acquired immunodeficiency syndrome (AIDS) or HIV infection, and allergic diathesis (including asthma).

Chronic obstructive pulmonary disease (COPD) included all cases categorised as chronic pulmonary diseases. Unspecified cardiac conditions were categorised under rheumatic heart disease, while uveitis was not included, as it was not considered a comorbidity in this context.

All patients were included in the analysis as complete data were available for most variables. The only exception was the duration of illness, for which 52 values were missing. These missing values were imputed using the mean value of the available data for that variable.

### Statistical analysis

#### Descriptive analysis

Patients were categorised into two groups: those with comorbidities and those without. For age-specific incidence analysis, patients were further stratified into two age groups: >50 years and ≤50 years. Descriptive statistics were used, with categorical variables presented as percentages and continuous variables as mean ± standard deviation (SD). Statistical analyses were performed using SPSS version 29.0.2.0. Fisher’s exact test and chi-square test were applied to categorical variables, while the two-tailed t-test was used for continuous variables. Data organisation and table generation were conducted using Microsoft® Excel® 2019 (Version 2409, Build 16.0.18025.20030) 64-bit. P-value <0.05 was considered statistically significant.

### Regression analysis

Comorbidities meeting two criteria were included in regression analysis: prevalence >1% in the study population and statistical significance in age group distribution. Logistic regression analyses were conducted to examine the associations between demographic factors and common comorbidities. The dependent variable was specified separately for rheumatological disease, hyperlipidaemia, diabetes, hypothyroid/hyper-thyroid conditions, infection, and hypertension. Covariates included age, gender, and duration of illness. Gender was treated as a categorical covariate, with female serving as the reference category.

The statistical model was configured with specific parameters to ensure analytical rigor. The probability thresholds were set at 0.05 for stepwise entry and 0.10 for removal. A classification cutoff of 0.5 was implemented, and the maximum number of iterations was set at 20. Multiple diagnostic measures were incorporated into the analysis. These included classification plots, Hosmer-Lemeshow goodness-of-fit testing, and case-wise listing of residuals, with identification of cases exceeding two standard deviations. The model calculated 95% confidence intervals for Exp(B). The final results were extracted from the “Variables in the Equation” table, which provided the Wald statistic and corresponding significance values.

## RESULTS

The study included 1,250 patients with SpA who met the ASAS criteria.^6^ The mean age of participants was 39.82 ± 13.28 years (range: 13–80 years). The female-to-male ratio was 0.45:1, with 387 females (30.96%) and 863 males (69.04%). The average duration of SpA at recruitment, based on data available from 1,149 patients, was 113.51 ± 89.72 months (range: 1–672 months). Among the participants, 937 (74.96%) had no comorbidities, while 313 (25.04%) had at least one comorbidity. The distribution of comorbidity counts showed that 18.88% had one comorbidity, 5.28% had two, 0.8% had three, and only 0.08% had four. Participants with comorbidities were older (mean age 49.19 ± 12.14 vs. 36.68 ± 12.13 years) and had a longer disease duration (132.06 ± 89.28 vs. 107.53 ± 89.09 months) (P < 0.001) (**Supplementary Table 1**).

**Table 1.** Distribution of comorbidities noted in the study population.

**Types of comorbidities**	**n (%)**
Hypertension	148 (11.84%)
Diabetes	106 (8.48%)
Hypothyroid/hyperthyroid	53 (4.24%)
Hyperlipidaemia	17 (1.36%)
Infection	17 (1.36%)
Rheumatologic disease	16 (1.28%)
Allergic asthma	11 (0.88%)
Cardiac disease	9 (0.72%)
Psychiatric disorder	6 (0.48%)
Neurological disorder	5 (0.4%)
Chronic pulmonary disease	3 (0.24%)
Hypotension	2 (0.16%)
Moderate-to-severe renal disease	1 (0.08%)
Recurrent diarrhoea (unknown cause)	1 (0.08%)
Inflammatory bowel disease	1 (0.08%)
Gout	1 (0.08%)
DVT	1 (0.08%)
Gastritis	1 (0.08%)
Dermatographic urticaria	1 (0.08%)
Glaucoma	1 (0.08%)
NASH	1 (0.08%)

Data is presented as a number (Percentage).

NASH: non-alcoholic steatohepatitis; DVT: deep vein thrombosis; TIA: transient ischemic attack.

The most prevalent comorbidity was hypertension (11.84%), followed by diabetes (8.48%), including 1.12% with chronic complications. Thyroid disorders were present in 4.24% of participants, while hyperlipidaemia and infections were each observed in 1.36%. Rheumatologic diseases were noted in 1.28%, including 0.64% with unspecified rheumatologic conditions, 0.4% with osteoarthritis, and 0.24% with psoriasis. The distribution of comorbidities noted in the study population is listed in **Table 1**. The detailed distribution of comorbidities is provided in **Supplementary Table 1**.

### Demographic and comorbid profile of participants based on age groups

Participants aged 50 years or younger had a significantly lower prevalence of comorbidities compared to those older than 50 years, with rates of 16.58% and 55.31%, respectively (P < 0.001). Additionally, there was a notable difference in gender distribution; the younger group had a higher proportion of males, with 720 males aged 50 years or younger compared to 143 males aged over 50 years (P < 0.001). The mean duration of SpA was significantly shorter in the younger group compared to the older group (99.93 ± 78.39 months vs. 159.70 ± 108.65 months; P < 0.001).

In the study, 83.42% of participants aged 50 years or younger had no comorbidities, compared to only 44.69% of those older than 50 (P < 0.001). The younger group showed a lower prevalence of comorbidities, with 13.41% having one comorbidity and 2.97% having two, compared to 38.46% and 13.55%, respectively, in the older group. Additionally, only 0.2% of the younger group had three comorbidities, versus 2.93% in the older group. Moreover, 0.37% of participants over 50 years had four comorbidities, an observation not seen in the younger group (**Table 2**).

**Table 2.** Comparison of demographic profiles and comorbidity patterns in SpA patients aged ≤50 years and >50 years.

**Characteristics**	**Less than or equal to 50 years (n = 977)**	**>50 years (n = 273)**	**P value**
**Demographic**			
Gender [F(M)]	257 (720)	130 (143)	<0.001*
Duration of illness (months)	99.93±78.39 (1–456)	159.70±108.65 (2–672)	<0.001#
**Comorbidity status**			
Present	162 (16.58%)	151 (55.31%)	<0.001*
Not present	815 (83.42%)	122 (44.69%)	
**Distribution of comorbidities**			
Cardiac disease	6 (0.61%)	3 (1.1%)	0.419^
Neurological disorder	3 (0.31%)	2 (0.73%)	0.301^
Chronic pulmonary disease	2 (0.2%)	1 (0.37%)	0.523^
Allergic asthma	8 (0.82%)	3 (1.1%)	0.713^
Rheumatologic disease	13 (1.33%)	3 (1.1%)	1.000^
Recurrent diarrhoea (unknown cause)	0 (0%)	1 (0.37%)	0.218^
Inflammatory bowel disease	1 (0.1%)	0 (0%)	1.000^
Gout	1 (0.1%)	0 (0%)	1.000^
Hyperlipidaemia	9 (0.92%)	8 (2.93%)	0.011*
Diabetes	39 (3.99%)	67 (24.54%)	<0.001*
Moderate-to-severe renal disease	0 (0%)	1 (0.37%)	0.218^
Hypothyroid/hyperthyroid	27 (2.76%)	26 (9.52%)	<0.001*
Infection	14 (1.43%)	3 (1.1%)	1.000^
Psychiatric disorder	6 (0.61%)	0 (0%)	0.349^
DVT	1 (0.1%)	0 (0%)	1.000^
Gastritis	1 (0.1%)	0 (0%)	1.000^
Dermatographic urticaria	1 (0.1%)	0 (0%)	1.000^
Glaucoma	1 (0.1%)	0 (0%)	1.000^
NASH	1 (0.1%)	0 (0%)	1.000^
Hypotension	2 (0.2%)	0 (0%)	1.000^
Hypertension	59 (6.04%)	89 (32.6%)	<0.001*
**Comorbidity count**			
0	815 (83.42%)	122 (44.69%)	<0.001^
1	131 (13.41%)	105 (38.46%)	
2	29 (2.97%)	37 (13.55%)	
3	2 (0.2%)	8 (2.93%)	
4	0 (0%)	1 (0.37%)	

Data is presented as mean ± SD (range) or number (Percentage).

P-value: *Chi-square test,

^ Fisher’s exact test,

# Two-tailed t-test. NASH: non-alcoholic steatohepatitis; DVT: deep vein thrombosis.

Several specific comorbidities were significantly less common in the younger age group (≤50 years), including diabetes (3.99% vs. 24.54%; P < 0.001), hypertension (6.04% vs. 32.6%; P < 0.001), and thyroid disorders (2.76% vs. 9.52%; P < 0.001), compared to the older group (>50 years). Furthermore, chronic conditions such as hyperlipidaemia, chronic pulmonary disease, diabetes with chronic complications, and cardiovascular disorders were also more frequently observed in participants aged >50 years (**Table 2**). The detailed distribution of comorbidities is provided in **Supplementary Table 2**.

### Demographic factors associated with comorbidity risk

Logistic regression analysis revealed significant associations between demographic factors and various comorbidities. Age emerged as the strongest predictor, showing significant associations with hyperlipidaemia (P = 0.022), diabetes (P < 0.001), thyroid disorders (P = 0.003), and hypertension (P < 0.001). Gender was significantly associated primarily with thyroid disorders (P < 0.001), while the duration of illness showed a significant relationship only with diabetes (P = 0.022). The strongest associations were observed between age and hypertension (Wald = 101.345), and between gender and thyroid disorders (Wald = 31.373) (**Table 3**). Detailed results are provided in **Supplementary Table 3**.

**Table 3.** Logistic regression analysis of demographic variables associated with comorbidities.

**Comorbidities**	**Age**		**Gender**		**Duration of illness**		**Constant**	
	Wald Statistic Score	Sig.	Wald Statistic Score	Sig.	Wald Statistic Score	Sig.	Wald Statistic Score	Sig.
**Rheumatologic disease**	0.667	0.414	0.852	0.356	1.52	0.218	9.6	0.002
**Hyperlipidaemia**	5.238	0.022	1.253	0.263	0.446	0.504	39.503	<0.001
**Diabetes**	82.871	<0.001	0.017	0.896	5.209	0.022	135.523	<0.001
**Hypothyroid/hyperthyroid**	8.865	0.003	31.373	<.001	0.101	0.751	40.894	<0.001
**Infection**	0.143	0.706	0	0.994	1.372	0.241	0	0.991
**Hypertension**	101.345	<0.001	0.897	0.344	0.073	0.787	163.256	<0.001

## DISCUSSION

According to the current study, approximately one in four patients with SpA had at least one comorbidity, highlighting the significant burden of concurrent conditions in this population. This finding is comparable to international data. For instance, in the Etude et Suivi des Polyarthrites Indifférenciées Récentes (ESPOIR) and Devenir des Spondylarthropathies Indifférenciées Récentes (DESIR) cohorts, 20.3% of SpA patients had at least one documented comorbidity.^11^ A study by Nikiphorou et al. reported a higher prevalence, with 51% of patients having at least one comorbidity and 9% presenting with three or more comorbidities.^12^ Similarly, a retrospective study of Indian population by Mahajan et al. reported that 48% of patients with AS had at least one comorbidity, notably higher than the present findings.^13^ These variations in the studies could be due to differences in study populations, methodologies, and definitions of comorbidities. The consistent presence of comorbid conditions across both national and international cohorts underscores the need for their routine assessment and integrated management in patients with SpA.

Comorbidity prevalence increased significantly with age, with patients over 50 years exhibiting a 3.3-fold higher rate of comorbidities compared to those aged 50 years or younger. Older individuals also showed a substantially higher prevalence of multiple coexisting conditions. The most frequently observed comorbidities included hypertension, diabetes (including diabetes with complications), thyroid disorders, hyperlipidaemia, and other rheumatologic diseases. These findings are consistent with those of a cross-sectional study by Yadav et al., conducted at a tertiary care hospital in India, which examined comorbidity patterns among patients with rheumatic diseases, including SpA. Among 224 SpA patients, hypertension (18.7%) was the most common comorbidity, followed by diabetes mellitus (13.3%) and hypothyroidism (9.3%). Their age-stratified analysis similarly demonstrated a progressive increase in comorbidity burden with advancing age: 76.4% in patients ≥60 years, 50.2% in those aged 40–59 years, and 25% in the 20–39 years group. This trend reinforces the association between increasing age and higher comorbidity burden in patients with SpA, emphasising the importance of age-tailored comorbidity screening and management strategies in clinical practice.^14^

Several previous studies have also reported similar findings, reinforcing the burden of comorbidities in SpA. A systematic review and meta-analysis by Zhao et al., which included 40 studies with a combined sample size of 119,427 patients with axial spondyloarthritis (axSpA), identified hypertension (22.3%), infections (18.3%), and hyperlipidaemia (17.1%) as the most prevalent comorbidities. The analysis further demonstrated that nearly all comorbidities were more common in ax-SpA patients compared to control populations.^15^ Mahajan et al. also reported hypertension as the most common comorbidity, present in 39% of patients.^13^ Similarly, the international ASAS-COMOSPA study found hypertension (34%) to be the most prevalent cardiovascular risk factor among SpA patients.^6^ Supporting these observations, a study by Bautista-Molano et al. reported arterial hypertension as one of the most frequent comorbidities in SpA, with a prevalence of 25.3% (95% CI: 21.2–30.0) and a significantly increased standardised risk ratio of 1.5 compared to the general population.^16^

Comparison of the prevalence of key comorbidities observed in the present cohort with their prevalence in the general Indian population, as reported in large-scale epidemiological studies, may aid in the meaningful interpretation of disease burden, healthcare needs, and priorities in the care of patients with spondyloarthritis. In a multicentric cross-sectional study by Babu et al., the overall prevalence of type 2 diabetes, based on random blood glucose measurements, was 4.77% (95% CI: 4.33–5.25) and based on fasting blood glucose, the prevalence was 6.80% (95% CI: 5.95–7.74). The prevalence of type 2 diabetes was found to be significantly associated with increasing age.^17^ Mohammad et al., using data from the fifth National Family Health Survey, reported an overall hypertension prevalence of 22.6% in India. The prevalence was higher among men (24.1%) compared to women (21.2%). Notably, the prevalence increased with age, reaching 48.4% in individuals aged 60 years and above.^18^ A retrospective study by Bansal et al., conducted at a tertiary care center, reported an overall prevalence of thyroid dysfunction of 24%. Hypothyroidism was the most common disorder, accounting for 51% of cases, followed by hyperthyroidism at 26%. Females were predominantly affected, comprising 68% of the cases, with the highest prevalence observed in the 18–35-year age group.^19^ The Indian Council of Medical Research–India Diabetes study reported significant regional variations in the prevalence of hypercholesterolemia (≥200 mg/dL), ranging from 4.6% to 50.3%. Higher rates were observed in the northern states, as well as in Kerala, Goa, and West Bengal.^20^

The current study findings of markedly different comorbidity patterns between younger and older patients are consistent with broader findings in SpA research. The higher comorbidity burden in older individuals aligns with results reported by Mahajan et al., where age-related conditions significantly influenced patient outcomes and added to the complexity of care. In their study, hypertension was the most common comorbidity (39.1%), followed by diabetes (26%), a pattern similar to our findings.^13^ Likewise, a study by Redeker et al., involving 1,776 patients with axSpA (mean age: 56.1 years), found that 41% had 1–2 comorbidities, 25% had 3–4, and 17% had five or more. Compared to a matched non-axSpA cohort (N = 8,880), hypertension was the most prevalent condition in both groups, but significantly higher among axSpA patients (52% vs. 38%). Other comorbidities, such as hypothyroidism (14% vs. 9%) and diabetes (16% vs. 13%), were also more frequently observed in the axSpA group.^7^

In the present study, a higher prevalence of comorbidities was observed in individuals over 50 years of age, and regression analysis confirmed that the likelihood of comorbidities increases with age. Conditions such as hypertension, diabetes, and thyroid disorders were more prevalent in the older age group compared to younger individuals. This observation is consistent with findings from the Biologic and Targeted Synthetic Antirheumatic Drugs Registry (BioStar), which reported hypertension (13.4%), diabetes mellitus (6.7%), and thyroid disorders (5.6%) as the most common comorbidities in SpA patients. In this study involving 1,242 patients, the comorbidity burden was significantly higher among those older than 60 years, with hypertension (55.9% vs. 10.0%, P <0.001), diabetes mellitus (28.0% vs. 5.0%, P <0.001), and thyroid dysfunction (10.5% vs. 5.2%, P=0.039) more frequently observed in the older group.^21^ This finding carries significant clinical implications, highlighting the need for more comprehensive screening protocols and closer monitoring of comorbid conditions in older SpA patients.

The gender distribution in the present study, with a majority of males, aligns with some previous studies.^16,21,22^ However, the strong association between female gender and thyroid disorders, suggests specific gender-related comorbidity patterns that warrant further investigation. Lange et al. reported a high prevalence of thyroid antibodies in women with AS.^23^ Çay et al. found thyroid dysfunction more prevalent in females (12.6%) compared to males (2.3%, P<0.001).^21^ Pawar et al. observed higher rates of thyroid dysfunction, especially hypothyroidism, in patients with systemic autoimmune disorders, highlighting the need for concurrent screening in women with these conditions.^24^

In the present study, regression analysis revealed a significant association between longer disease duration and the presence of diabetes. Similarly, Derakhshan et al., using data from the ASAS-COMOSPA cohort, found that prolonged SpA disease duration was significantly associated with an increased risk of hypertension. Specifically, for every five-year increase in disease duration, the odds of developing hypertension rose by 12.9% (OR 1.129, 95% CI 1.072–1.189; P < 0.001).^25^ Furthermore, a Mendelian randomisation analysis by Ren et al. demonstrated a significant association between AS and an elevated risk of both type 1 and type 2 diabetes mellitus.^26^ A systematic review and meta-analysis that included 23 studies and a total of 65,025 patients reported a pooled diabetes prevalence of 7.0% among individuals with axSpA (95% CI: 5.9–8.0%; predictive interval: 2.4–12.9%; P < 0.001). These findings indicate a higher prevalence and a potential increased risk of diabetes in individuals with axSpA.^27^ These findings suggest a higher prevalence and potentially increased risk of diabetes in individuals with axSpA. Therefore, routine screening for diabetes and the promotion of lifestyle modifications should be integral components of care in this patient population.

The study holds significant relevance in the Indian context, given the limited research on the prevalence of comorbidities in SpA patients in the country. However, reliance on patient-reported data and medical charts introduces a potential risk of underreporting or mis-classification. Despite this limitation, the study offers several strengths that enhance its clinical relevance and applicability. It underscores the need for age-specific approaches to comorbidity screening in SpA patients to ensure timely detection and management. Additionally, it highlights the importance of comprehensive cardiovascular risk assessment, particularly in older individuals, and suggests that gender-specific monitoring may be beneficial, especially for conditions such as thyroid disorders in female patients.

The key strength of this study lies in its broad geographic representation of the patient population, with data collected from seven centres across India, thereby enhancing the generalisability of its findings. The use of a standardised pro forma and the provision of training for CRAs and PIs ensured consistency and accuracy in data collection. The large sample size enabled robust statistical analysis of comorbidities. Future research should explore the temporal relationship between SpA and its comorbidities and assess whether early intervention in younger patients can reduce long-term comorbidity risk. Additionally, studies examining the impact of comorbidities on SpA outcomes could further inform and improve patient care.

While several publications in the *Mediterranean Journal of Rheumatology* have explored various clinical, therapeutic, and extra-articular aspects of SpA, data specifically addressing the prevalence and predictors of comorbidities in the Indian population remain limited. Most existing studies, such as those focusing on extra-articular manifestations, treatment responses, or diagnostic challenges, have not systematically evaluated comorbid conditions or their demographic and clinical determinants. The current study adds to the existing literature by providing multicentre, real-world data from India, identifying age, gender, and disease duration as key determinants of comorbidity burden in SpA. This addresses a significant knowledge gap by providing region-specific insights into the epidemiology of comorbidities in SpA, which are essential for guiding screening, risk stratification, and holistic patient management in the Indian context.

## CONCLUSION

The study highlights the considerable comorbidity burden in SpA patients, with hypertension, thyroid disorders, and diabetes being the most prevalent. Older age, longer disease duration, and female gender were found to be associated with a higher prevalence of comorbidities. These findings emphasise the importance of developing tailored treatment and management strategies to enhance outcomes for older SpA patients dealing with multiple chronic conditions.

## APPENDIX

### Appendix

**Supplementary Table 1.** Descriptive characteristics of participants with and without co-morbidities (n = 1250).

**Characteristics**	**Overall descriptive (n = 1250)**	**Co-morbidities absent (n = 937)**	**Co-morbidities present (n = 313)**	**P-value**
**Age**	39.82±13.28 (13–80)	36.68±12.13 (13–80)	49.19±12.14 (16–76)	<0.001#
**Gender (F/M)**	387 (863)	251 (686)	136 (177)	<0.001*
**Duration of illness (months)**	113.51±89.72 (1–672)	107.53±89.09 (1–672)	132.06±89.28 (1–432)	<0.001#
**Co-morbidity count**				
0	937 (74.96%)			
1	236 (18.88%)			
2	66 (5.28%)			
3	10 (0.8%)			
4	1 (0.08%)			
**Distribution of comorbidities**				
**Types of comorbidities**			n (%)	
**Diabetes**				
**Overall**			106 (8.48%)	
**Diabetes**			92 (7.36%)	
**Diabetes with chronic complications**			14 (1.12%)	
**Infection**				
**Overall**			17 (1.36%)	
**Tuberculosis**			8 (0.64%)	
**Hepatitis B**			8 (0.64%)	
**Hepatitis C**			1 (0.08%)	
**Rheumatologic disease**				
**Overall**			16 (1.28%)	
**Rheumatologic disease**			8 (0.64%)	
**Psoriasis**			3 (0.24%)	
**Osteoarthritis**			5 (0.4%)	
**Psychiatric disorder**				
**Overall**			6 (0.48%)	
**Psychiatric disease**			1 (0.08%)	
**Depression**			2 (0.16%)	
**Nerve problems**			2 (0.16%)	
**Anxiety**			1 (0.08%)	
**Neurological disorder**				
**Overall**			5 (0.4%)	
**Migraine**			2 (0.16%)	
**Cerebrovascular disease (Stroke, transient ischemic attack)**			2 (0.16%)	
**Epilepsy**			1 (0.08%)	
**Cardiac disease**				
**Overall**			9 (0.72%)	
**Prior myocardial infarction**			6 (0.48%)	
**Rheumatic heart disease**			3 (0.24%)	

Data is presented as mean ± SD (range) or number (Percentage).

P value: *chi-square,

# t-test.

**Supplementary Table 2.** Comparison of comorbidity patterns in Spondyloarthritis patients aged ≤50 years and >50 years.

**Distribution of comorbidities**	**Less than or equal to 50 (n = 977)**	**Above 50 (n = 273)**	**P value**
**Cardiac disease**			
**Overall**	6 (0.61%)	3 (1.1%)	0.419^
**Prior myocardial infraction**	3 (0.31%)	3 (1.1%)	0.122^
**Rheumatic heart disease**	3 (0.31%)	0 (0%)	1.000^
**Neurological disorder**			
**Overall**	3 (0.31%)	2 (0.73%)	0.301^
**Migraine**	2 (0.2%)	0 (0%)	1.000^
**Cerebrovascular disease (stroke, transient ischemic attack)**	0 (0%)	2 (0.73%)	0.048^
**Epilepsy**	1 (0.1%)	0 (0%)	1.000^
**Rheumatologic disease**			
**Overall**	13 (1.33%)	3 (1.1%)	1.000^
**Rheumatologic disease**	8 (0.82%)	0 (0%)	0.212^
**Psoriasis**	2 (0.2%)	1 (0.37%)	0.523^
**Osteoarthritis**	3 (0.31%)	2 (0.73%)	0.301^
**Diabetes**			
**Overall**	39 (3.99%)	67 (24.54%)	<0.001*
**Diabetes**	36 (3.68%)	56 (20.51%)	<0.001*
**Diabetes with chronic complications**	3 (0.31%)	11 (4.03%)	<0.001^
**Infection**			
**Overall**	14 (1.43%)	3 (1.1%)	1.000^
**Tuberculosis**	6 (0.61%)	2 (0.73%)	0.688^
**Hepatitis B**	8 (0.82%)	0 (0%)	0.212^
**Hepatitis C**	0 (0%)	1 (0.37%)	0.218^
**Psychiatric disorder**			
**Overall**	6 (0.61%)	0 (0%)	0.349^
**Psychiatric disease**	1 (0.1%)	0 (0%)	1.000^
**Depression**	2 (0.2%)	0 (0%)	1.000^
**Nerve problems**	2 (0.2%)	0 (0%)	1.000^
**Anxiety**	1 (0.1%)	0 (0%)	1.000^

Data is presented as mean ± SD (range) or number (Percentage).

P-value: * Chi-square test,

^ Fisher's exact test,

# Two-tailed t-test.

**Supplementary Table 3.** Logistic regression analysis of demographic predictors for comorbidities.

**Comorbidities**			**B**	**S.E.**	**Wald**	**df**	**Sig.**	**Exp(B)**	**95% C.I. for EXP(B)**	
									**Lower**	**Upper**
**Rheumatologic disease**	**Step 1**	Age	−0.022	0.027	0.667	1	0.414	0.978	0.928	1.031
		Gender	0.734	0.795	0.852	1	0.356	2.083	0.438	9.896
		Duration of illness	−0.006	0.005	1.52	1	0.218	0.994	0.984	1.004
		Constant	−3.748	1.21	9.6	1	0.002	0.024		
**Hyperlipidaemia**	**Step 1**	Age	0.045	0.02	5.238	1	0.022	1.046	1.006	1.087
		Gender	0.673	0.601	1.253	1	0.263	1.96	0.603	6.37
		Duration of illness	0.002	0.002	0.446	1	0.504	1.002	0.997	1.006
		Constant	−6.927	1.102	39.503	1	<.001	0.001		
**Diabetes**	**Step 1**	Age	0.081	0.009	82.871	1	<.001	1.085	1.066	1.104
		Gender	−0.03	0.226	0.017	1	0.896	0.971	0.623	1.512
		Duration of illness	−0.003	0.001	5.209	1	0.022	0.997	0.995	1
		Constant	−5.651	0.485	135.523	1	<.001	0.004		
**Hypothyroid/Hyperthyroid**	**Step 1**	Age	0.036	0.012	8.865	1	0.003	1.037	1.012	1.062
		Gender	−2.241	0.4	31.373	1	<.001	0.106	0.049	0.233
		Duration of illness	−0.001	0.002	0.101	1	0.751	0.999	0.996	1.003
		Constant	−3.727	0.583	40.894	1	<.001	0.024		
**Infection**	**Step 1**	Age	0.016	0.043	0.143	1	0.706	1.016	0.935	1.105
		Gender	15.943	2051.834	0	1	0.994	8396263.593	0	.
		Duration of illness	0.005	0.004	1.372	1	0.241	1.005	0.997	1.013
		Constant	−22.568	2051.834	0	1	0.991	0		
**Hypertension**	**Step 1**	Age	0.083	0.008	101.345	1	<.001	1.087	1.069	1.105
		Gender	−0.191	0.202	0.897	1	0.344	0.826	0.556	1.227
		Duration of illness	0	0.001	0.073	1	0.787	1	0.998	1.002
		Constant	−5.675	0.444	163.256	1	<.001	0.003		
